# The Prognostic Impact of TGF-β1, Fascin, NF-κB and PKC-ζ Expression in Soft Tissue Sarcomas

**DOI:** 10.1371/journal.pone.0017507

**Published:** 2011-03-03

**Authors:** Andrej Valkov, Sveinung W. Sorbye, Thomas K. Kilvaer, Tom Donnem, Eivind Smeland, Roy M. Bremnes, Lill-Tove Busund

**Affiliations:** 1 Department of Clinical Pathology, University Hospital of Northern Norway, Tromsø, Norway; 2 Institute of Medical Biology, University of Tromsø, Tromsø, Norway; 3 Department of Oncology, University Hospital of Northern Norway, Tromsø, Norway; 4 Institute of Clinical Medicine, University of Tromsø, Tromsø, Norway; City of Hope National Medical Center and Beckman Research Institute, United States of America

## Abstract

**Aims:**

Transforming growth factor-β (TGF-β), fascin, nuclear factor-kappa B (NF-κB) p105, protein-kinase C-zeta (PKC-ζ), partioning-defective protein-6 (Par-6), E-cadherin and vimentin are tumor promoting molecules through mechanisms involved in cell dedifferentiation. In soft tissue sarcomas, their expression profile is poorly defined and their significance is uncertain. We aimed to investigate the prognostic impact of TGF-β1, NF-κB p105, PKC-ζ, Par-6α, E-cadherin and vimentin in non-gastrointestinal stromal tumor soft tissue sarcomas (non-GIST STSs).

**Patients and Methods:**

Tumor samples and clinical data from 249 patients with non-GIST STS were obtained, and tissue microarrays (TMAs) were constructed for each specimen. Immunohistochemistry (IHC) was used to evaluate marker expression in tumor cells.

**Results:**

In univariate analysis, the expression levels of TGF-β1 (P = 0.016), fascin (P = 0.006), NF-κB p105 (P = 0.022) and PKC-ζ, (P = 0.042) were significant indicators for disease specific survival (DSS). In the multivariate analysis, high TGF-β1 expression was an independent negative prognostic factor for DSS (HR = 1.6, 95% CI = 1.1–2.4, P = 0.019) in addition to tumor depth, malignancy grade, metastasis at diagnosis, surgery and positive resection margins.

**Conclusion:**

Expression of TGF-β1 was significantly associated with aggressive behavior and shorter DSS in non-GIST STSs.

## Introduction

Soft tissue sarcomas (STS) are malignant tumors arising from extraskeletal connective tissues. They are group of heterogeneous neoplasms, consisting of more than 50 subtypes, but comprise less than 1% of adult malignancies [1], [2]. Approximately 50% of the STS patients will succumb to their disease because of metastasis or local relapse [3]. The prognostic factors determining tumor progression and ultimately patients' fate include tumor grade, size, location, depth, histological entity, positive resection margins and presence of local recurrence [4]–[10]. Much attention is also paid to recurrent gene aberrations in STSs as the predictive biomarkers [11]–[13].

Molecular mechanisms regulating tissue changes from benign to invasive and finally to metastatic neoplasia is an area of growing scientific interest. Malignant transformation in epithelial tumors is described as epithelial-to-mesenchymal transition (EMT). EMT is defined as a sequence of protein modifications and transcriptional events in response to a certain set of extracellular stimuli leading to a stable, but sometimes reversible, cellular change [14].

Multiple molecular mediators of EMT have been described in carcinomas [15]. The list of EMT pathways includes nuclear factor-kappa B (NF-κB), AKT/mammalian target of rapamycin (AKT/mTOR) axis, mitogen-activated protein kinase (MAPK), beta-catenin, protein-kinase C (PKC) and others [16]. However, expression of markers linked to EMT does not support EMT as a biological event in STSs. Moreover, the markers linked to EMT have clearly defined roles in tumor biology that are distinct from EMT, and the negative impact of these factors on tumor behavior can be rather defined as “defifferentiation” or “anaplasia” in these tumors. The NF-κB and TGF-β pathways have been described to influence the prognosis in several types of STS, including malignant fibrous histiocytoma, Ewing sarcoma, osteosarcoma and rhabdomyosarcoma [17]–[21]. Nevertheless, there are no reports with emphasis on the prognostic value of E-cadherin, fascin, Par-6 and PKC-ζ in STS. Vimentin, which is by definition expressed by all STS, is a classic marker of higher aggressivity in carcinoma. The intensity of vimentin expression can fluctuate, and the significance of this variation for the STS patients' survival is not clear.

In this study, we investigate the expression of a panel of seven molecular biomarkers in 249 non-GIST STS patients. We realize that these tumors belong to different histological subtypes and consequently have diverse prognoses. However, they all have mesenchymal derivation and belong therefore to the same generic group, STS. The investigated dedifferentiation markers reflect universal and basic processes in tumorigenesis, they are described in a variety of epithelial and non-epithelial tumors of different locations and histological entities and seem to not depend on tumor type. This is confirmed by the fact that almost each of STS type we investigated can show broad spectrum of malignancy grade, from almost benign to high grade malignant tumor.

To our knowledge this is the first evaluation of such large collection of dedifferentiation-associated biomarkers in non-GIST STSs related to DSS.

## Materials and Methods

### Patients and clinical samples

Primary tumor tissue from anonymized patients diagnosed with non-GIST STS at the University Hospital of Northern Norway (UNN) 1973–2006 and The Hospitals of Arkhangelsk region, Russia, were used in this retrospective study. In total, 496 patients were registered from the hospital databases. Of these, 247 patients were excluded due to missing clinical data (n = 86) or inadequate material for histological examination (n = 161). Thus, 249 STS patients with full clinical records and adequate paraffin-embedded tissue blocks were eligible.

This report includes follow-up data as of September 2009. The median follow-up was 38 months (range 0.1–392). Formalin-fixed and paraffin-embedded tumor specimens were obtained from the archives of the Departments of Pathology at UNN and the Arkhangelsk hospitals. The tumors were graded according to the French Fèdèration Nationales des Centres de Lutte Contre le Cancer (FNCLCC) [22].

### Microarray construction

All sarcomas were histologically reviewed by two trained pathologists (S.S. and A.V.) and the most representative areas of viable tumor cells (neoplastic cells) were carefully selected and marked on the hematoxylin and eosin (H&E)-stained slides and sampled for the tissue microarray blocks (TMAs). The TMAs were assembled using a tissue-arraying instrument (Beecher Instruments, Silver Springs, MD). The Detailed methodology has been previously reported [23]. Briefly, we used a 0.6 mm diameter stylet, and the study specimens were routinely sampled with two replicate core samples (different areas) of neoplastic tissue. To include all core samples, 12 tissue array blocks were constructed. Multiple 4-µm sections were cut with a Micron microtome (HM355S) and stained using specific antibodies for immunohistochemistry (IHC) analyses.

### Immunohistochemistry (IHC)

The applied antibodies were subjected to in-house validation by the manufacturer for IHC analysis on paraffin-embedded material. Fascin, 55K2; Cat.no. MAB3582 (mouse monoclonal; Chemicon International; 1∶25), NF-κB p105 (Ser933)178F3 Cat.no. 4808 (rabbit monoclonal; Cell Signaling Technology; 1∶50), TGF-β1 (V)∶sc-146 (rabbit polyclonal; Santa Cruz; 1∶50), PKC-ζ (C-20):sc-216 (rabbit polyclonal; Santa Cruz; 1∶100), Par-6α (H-90)∶sc-25525 (rabbit polyclonal; Santa Cruz; 1∶10) E-cadherin (mouse monoclonal; ECH-6; Cell Marque; prediluted), and vimentin (mouse monoclonal; V9; Ventana Medical Systems; prediluted).

Sections (4 µM) were deparaffinized with xylene and rehydrated with ethanol. Fascin and NF-kB were stained manually. Antigen retrieval was performed exposing slides to microwave heating for 20 min at 450 W in 0.01M Citrate buffer pH 6.0. Primary antibodies were incubated overnight in +4 degrees C (NF-kB), and for 30 min at room temperature (fascin). Visualization reagents were Vectastein ABC Elite-kit from Vector Laboratories (NF-kB) and Envision+System-HRP (DAB) from DAKO (fascin).

TGF-β1, PKC-ζ, Par-6α, E-cadherin and vimentin were stained using Ventana Benchmark XT (Ventana Medical Systems Inc), procedure iViewDAB. Antigen retrieval was CC1 mild (TGF-β1, PKC-ζ, Par-6α, E-Cadherin) and CC1 Standard (vimentin). For E-cadherin post-fixative was selected. Primary antibodies against TGF- β 1, PKC-ζ, E-cadherin and Par-6α were incubated at 37°C for 28, 28, 32 and 52 min, accordingly. As secondary antibodies biotinylated goat anti-mouse IgG and mouse anti-rabbit IgM were used. This was followed by application of liquid diaminobenzidine as substrate-chromogen, yielding a brown reaction product at the site of the target antigen (iView DAB® procedure). Finally, slides were counterstained with hematoxylin to visualize the nuclei. For each antibody, including negative controls, all TMA staining were performed in a single experiment.

### Scoring of IHC

**Figure 1:**
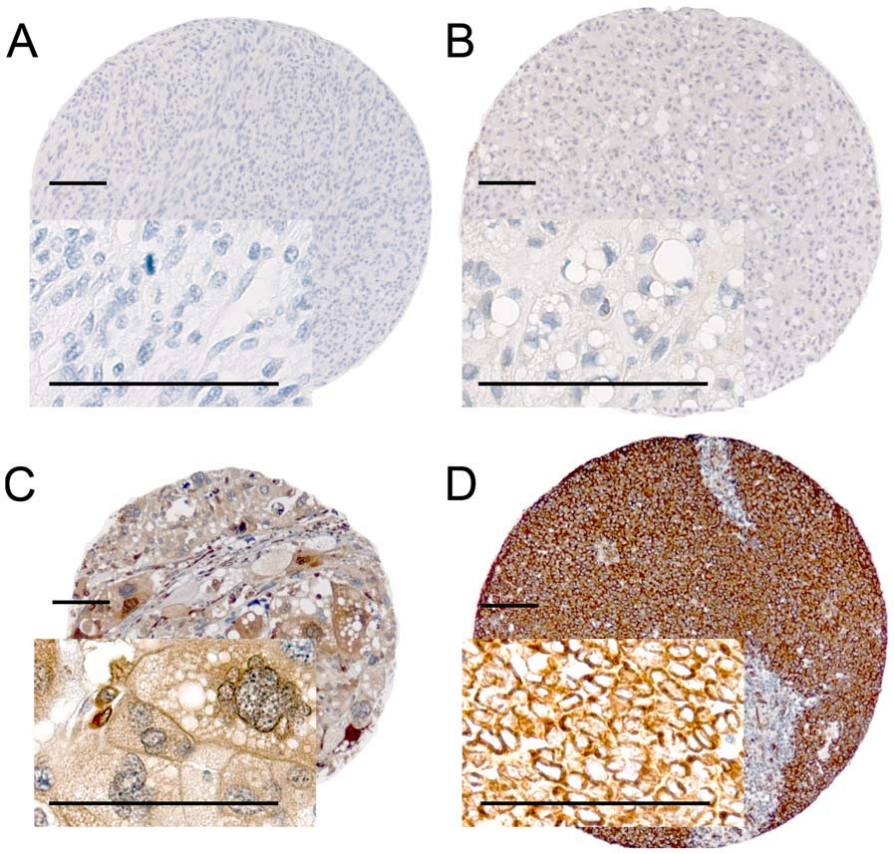
IHC analysis of TMA of non-GIST STS representing different scores of expression of dedifferentiation related markers in tumor cells. A, Leiomyosarcoma, histological grade I, E-cadherin, negative staining, score 0; B, Dedifferentiated liposarcoma, histological grade II, TGF-β1, weak staining, score 1; C, Pleomorphic liposarcoma, histological grade III, Fascin, moderate staining; score 2; D, Alveolar rhabdomyosarcoma, histological grade III, Vimentin, strong staining, score 3. All calibration bars correspond to 100 µm. Abbreviations; IHC, immunohistochemistry; TMA, tissue microarray; non-GIST STS, non gastro-intestinal stromal tumor soft-tissue sarcoma; TGF-β1, transforming growth factor beta 1.

The ARIOL imaging system (Genetix, San Jose, CA) was used to scan the slides with immunohistochemically stained TMAs. The specimens were scanned at a low resolution (1.25×) and high resolution (20×) using Olympus BX 61 microscope with an automated platform (Prior). The slides were loaded in the automated slide loader (Applied Imaging SL 50). Representative and viable tissue sections were scored manually on computer screen, semiquantitatively for cytoplasmic staining. The dominant staining intensity in neoplastic cells was scored subjectively as: 0 = negative; 1 = weak; 2 = intermediate; 3 = strong (Figure 1). All samples were anonymized and independently scored by two pathologists (A.V. and S.S.). In cases where score difference was equal or exceeding 2, the slides were re-examined and a consensus was reached by the observers. When assessing a score for a given core, the observers were blinded to the scores of the other variables and to outcome. Mean score for duplicate cores from each individual was calculated.

### Statistical methods

All statistical analyses were done using the statistical package SPSS (Chicago, IL), version 16. The IHC scores from each observer were compared for interobserver reliability by use of a two-way random effect model with absolute agreement definition. The intraclass correlation coefficient (reliability coefficient) was obtained from these results. The Chi-square test and Fishers Exact test were used to examine the association between molecular marker expression and various clinicopathological parameters. Univariate analyses were done by using the Kaplan-Meier method, and statistical significance between survival curves was assessed by the log rank test. Disease-specific survival (DSS) was determined from the date of histological confirmed STS diagnosis to the time of STS death. To assess the independent value of different pretreatment variables on survival, in the presence of other variables, multivariate analysis was performed using the Cox proportional hazards model. Only variables of significant value from the univariate analysis were entered into the Cox regression analysis. Probability for stepwise entry and removal was set at 0.05 and 0.10, respectively. The significance level used in both univariate multivariate analyses was P<0.05, but in the post hoc subgroup analysis the significance level was moved from P = 0.05 to P = 0.01 due to risk of false positivity.

### Ethical clearance

The National Cancer Data Inspection Board and The Regional Committee for Research Ethics approved the study. The Regional Committee approved that written consent from the patients for their information to be stored in the hospital database and used for research was not needed because most of the material was more than 20 years old and most of the patients are now dead. The material was collected from our approved biobank for paraffin-embedded material and slides. All material was anonymously collected. The data were analyzed anonymously.

## Results

### Clinicopathological variables

The clinicopathological variables are summarized in Table 1. Median age was 59 (range, 0–91) years and 56% were female. The non-GIST STS comprised 249 tumors including pleomorphic sarcoma (n = 68), leiomyosarcoma (n = 67), liposarcoma (n = 34), fibrosarcoma (n = 20), rhabdomyosarcoma (n = 16), synovial sarcoma (n = 16), angiosarcoma (n = 13), malignant peripheral nerve sheath tumor (MPNST) (n = 11) and other types of sarcoma (n = 4). The tumors were localized in the extremities (n = 89), trunk (n = 47), retroperitoneum (n = 37), head/neck (n = 18) and viscera (n = 58). The treatment option of choice was surgery (n = 228), 120 patients received surgery alone, 55 patients received surgery and radiotherapy, 40 patients received surgery and chemotherapy, 13 patients received surgery, radiotherapy and chemotherapy. Of the non-operated patients (inoperable, n = 11; advanced age/other serious disease, n = 5, STS diagnosis confirmed post mortem, n = 3; patient refusal, n = 2) seven received chemotherapy and/or radiotherapy. Fourteen patients did not obtain any treatment.

**Table 1:** Prognostic clinicopathological variables as predictors for disease-specific survival in 249 non-GIST STSs (univariate analyses, log-rank test).

Characteristic	Patients(n)	Patients(%)	Median survival(months)	5-Year survival(%)	P
**Age**					
≤ 20 years	20	8	15	40	0.126
21–60 years	113	45	68	52	
>60 years	116	47	30	40	
**Gender**					
Male	110	44	41	46	0.390
Female	139	56	45	45	
**Patient nationality**					
Norwegian	167	67	63	51	0.011
Russian	82	33	22	34	
**Histological entity**					
Pleomorphic sarcoma	68	27	29	40	0.102
Leiomyosarcoma	67	27	45	46	
Liposarcoma	34	14	NR	67	
MF/MFT	20	8	43	50	
Angiosarcoma	13	5	10	31	
Rhabdomyosarcoma	16	6	17	38	
MPNST	11	5	49	45	
Synovial sarcoma	16	6	31	29	
Other STSs	4	2	NR	18	
**Tumor localization**					
Extremities	89	36	100	53	0.348
Trunk	47	29	32	44	
Retroperitoneum	37	25	25	38	
Head/Neck	18	7	15	41	
Visceral	58	23	30	42	
**Tumor size**					
≤ 5 cm	74	30	127	57	0.027
5–10 cm	91	37	44	45	
>10 cm	81	32	28	36	
Missing	3	1			
**Malignancy grade**					
1	61	25	NR	74	<0.001
2	98	39	41	45	
3	90	36	16	26	
**Tumor depth**					
Superficial	17	7	NR	93	<0.001
Deep	232	93	36	42	
**Metastasis at time of diagnosis**					
No	206	83	76	53	<0.001
Yes	43	17	10	10	
**Surgery**					
Yes	228	92	59	50	<0.001
No	21	8	5	0	
**Resection margins**					
Free	178	71	127	66	<0.001
Not free/no surgery	71	29	10	18	
**Chemotherapy**					
No	191	77	52	47	0.424
Yes	58	23	29	40	
**Radiotherapy**					
No	176	71	48	46	0.590
Yes	73	29	38	43	

Abbreviations: NR, not reached; MF/MFT, malignant fibroblastic/myofibroblastic tumors; MPNST, malignant peripheral nerve sheath tumor; NOS, not otherwise specified; non-GIST STS, non-gastro intestinal stromal tumor soft-tissue sarcoma.

### Interobserver variability

Interobserver scoring agreement was tested for all markers. The intraclass correlation coefficients were as follows: 0.92 for E-cadherin (P<0.001), 0.89 for fascin (P<0.001), 0.91 for NF-κB p105 (P<0.001), 0.86 for Par-6α (P<0.001), 0.97 for PKC-ζ (P<0.001), 0.87 for TGF-β1 (P<0.001) and 0.93 for vimentin (P<0.001).

### Expression pattern and correlations with clinicopathological variables

The TGF-β1, NF-κB p105, fascin, Par-6α, PKC-ζ and vimentin, showed expression in the cytoplasm of tumor cells while E-cadherin demonstrated focal membrane-associated and/or cytoplasmic positivity in a minority of the tumors.

TGF-β1, fascin and Par-6α expression significantly correlated with STS histological grade. Low-grade tumors expressed TGF-β1 in 20% of cases, while high-grade tumors did so in 42% (P = 0.008). For fascin, this low- to high grade ratio of marker expression comprised 15% to 52% (P<0.001). PKC-ζ, Par-6α and NF-κB p105 positivity in STSs correlated with their subsequent metastatic behavior. PKC-ζ expression was observed in 36% of metastasizing tumors, whereas only 22% non-metastasizing STSs (P = 0.016) were PKC-ζ positive. For Par-6α this metastasizing versus non-metastasizing characteristic comprised 72% and 56% (P = 0.012), and for NF-κB p105, 85% and 69% (P = 0.005), respectively. None of the investigated markers correlated significantly with age, gender, tumor location, depth, size or relapse rate.

### Univariate analyses

Data are presented in Table 1. Patient nationality (P = 0.011), tumor size (P = 0.027), malignancy grade (P<0.001), tumor depth (P<0.001), metastasis at time of diagnosis (P<0.001), surgery (P<0.001) and resection margins (P<0.001) were all significant prognostic variables for DSS.

**Figure 2:**
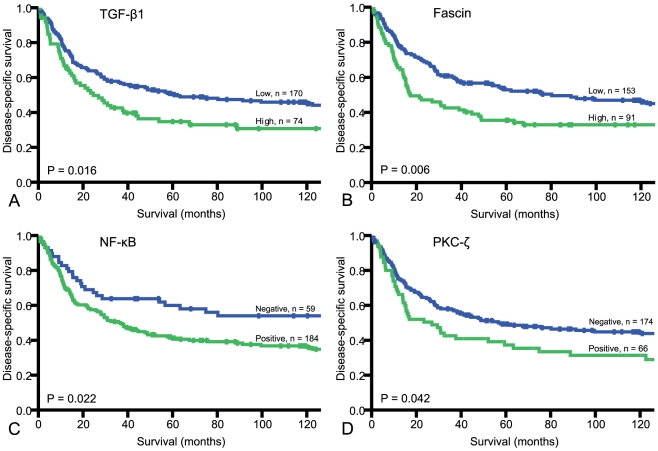
Disease-specific survival curves for dedifferentiation-associated markers. A, TGF-β1; B, Fascin; C, NF-κB p105; D, PKC-ζ. Abbreviations: TGF-β1, transforming growth factor beta 1; NF-κB, nuclear factor-κB; PKC-ζ, protein kinase C zeta.

The prognostic impact on DSS by the investigated molecular factors is shown in Table 2. Among these, TGF-β1 (P = 0.016), fascin (P = 0.006), NF-kB p105 (P = 0.022) and PKC-ζ (P = 0.042) were significant indicators of shorter DSS. Disease-specific survival curves for these markers are correspondingly shown in Figure 2, A–D.

**Table 2:** Tumor expression of markers associated with dedifferentiation and their prognostic impact on disease-specific survival in patients with non-GIST STSs (univariate analyses; log-rank test, n = 249).

Marker expression	Patients(n)	Patients(%)	Median survival(months)	5-Year survival(%)	P
**TGF-β1**					
Low	170	68	62	66	0.016
High	74	30	25	31	
Missing	5	2			
**E-cadherin**					
Negative	193	77	58	71	0.659
Positive	39	16	48	39	
Missing	17	7			
**Fascin**					
Low	153	61	80	53	0.006
High	91	37	17	36	
Missing	5	2			
**NF-κB p105**					
Negative	59	24	NR	60	0.022
Positive	184	74	37	41	
Missing	6	2			
**Par-6α**					
Low	91	37	62	50	0.283
High	153	61	38	44	
Missing	5	2			
**PKC-ζ**					
Negative	174	70	57	49	0.042
Positive	66	27	27	37	
Missing	9	3			
**Vimentin**					
Low	83	34	48	46	0.616
High	157	63	41	45	
Missing	9	3			

Abbreviations: Non-GIST STS, non-gastro intestinal stromal tumor soft-tissue sarcoma; TGF-β1, transforming growth factor beta 1; NF-κB, nuclear factor-κB; Par-6α, partitioning-defective protein 6α; PKC-ζ, protein kinase C zeta; NR, not reached.

Stratification of cases based on clinical variables revealed that high TGF-β1 expression was a negative prognostic indicator particularly for pleomorphic sarcoma (P<0.001) and for trunk-located STS (P = 0.003).

### Multivariate Cox proportional hazards analyses

Only variables which were significant in univariate analyses were entered into the multivariate analysis. The results of the multivariate analysis are presented in Table 3. Tumor depth (P = 0.017), histological entity (P = 0.027), malignancy grade (P<0.001), metastasis at time of diagnosis (P = 0.011), surgery (P = 0.002), non-free resection margins (P<0.001), and TGF-β1 expression (P = 0.035) were significant independent prognostic indicators of DSS.

**Table 3:** Results of the Cox regression analysis summarizing significant independent prognostic factors in the overall material.

Factor	Hazard Ratio	95% CI	P
**Tumor depth**			
Superficial	1.0		
Deep	9.6	1.3–69	0.025
**Malignancy grade**			<0.001*
1	1.0		
2	2.5	1.4–4.3	0.002
3	3.2	1.8–5.7	<0.001
**Metastasis at the time of diagnosis**			
No	1.0		
Yes	1.8	1.2–2.9	0.010
**Surgery**			
Yes	1.0		
No	2.7	1.4–5.3	0.002
**Resection-margins**			
Free	1.0		
Non-free	2.9	1.9–4.4	<0.001
**TGF-β1**			
Low	1.0		
High	1.6	1.1–2.4	0.019

Abbreviations: TGF-β1, transforming growth factor beta 1.

*Overall significance as a prognostic factor.

## Discussion

In our large-scale retrospective study we sought to investigate the prognostic impact of a set of biomarkers in non-GIST STS patients. These markers are known to participate in the process of EMT in epithelial tumors [14], but bear other important biological functions as well. Moreover, the EMT concept has not received general acceptance [24]. STSs are of mesenchymal origin and can demonstrate a range of behavior patterns, varying from almost benign to highly aggressive tumors. TGF-β1, fascin, NF-kB p 105 and PKC-ζ showed significant unfavorable influence on survival in the univariate analyses. Besides, high expression of TGF-β1 was a significant independent negative prognostic indicator of DSS. To our knowledge this is the first prognostic evaluation of these biomarkers in whole-array non-GIST STSs.

TGF-β is a multifunctional cytokine known to induce G1 arrest in order to end proliferation, induce differentiation, or promote apoptosis in normal cells, thus being a natural tumor-suppressive agent. Though in tumorigenesis this mediator initiates EMT through activation of Smad and non-Smad signalling pathways [25]. Such pro-neoplastic action becomes possible through either blockade of the TGF-β pathway with receptor-inactivating mutations, or selective inactivation of the tumor-inhibiting arm of this pathway [26]. Another possibility is TGF-β induced systemic immune suppression [27]. The TGF-β pathway activation has been shown to negatively influence prognosis both in epithelial [28], [29] and in mesenchymal bone [30] and soft tissue tumors [18], [21], [31]. The latter studies, however, are devoted to one particular STS type, while investigations of TGF-β1 expression by whole-array human STS with concern to impact on survival are not reported. In the present study, TGF-β1 was found to be a crucial prognostic marker. It had an independent significantly negative prognostic effect on DSS in non-GIST STS.

TGF-β was called the Jekyl and Hyde of cancer [32] for its ability to modulate its action from tumor promoter to tumor suppressor. The factors responsible for such transition remain unclear. The candidates are both tumour-cell-autonomous TGF-β signalling [33] itself, and factors in the tumor microenvironment. Among the latter, inflammatory cells and cancer-associated fibroblasts [27], as well as angiogenetic factors [33], are considered the most potent modulators of TGF-β action. In previous studies, we have investigated the prognostic value of both inflammatory cells [34] and angiogenetic factors in STSs [35], and further plan to explore their interactions with TGF-β.

Fascin and E-cadherin are both related to cell motility and cell adhesiveness and important factors in the progression and metastasis of cancers [36]. Fascin is reported to be overexpressed in sarcomatoid, in contrast to conventional, non-small cell lung carcinoma [37]. In leiomyomatous tumors of the uterus it was associated with higher malignancy grade [38]. We found fascin expression to be associated with a shorter STS survival in univariate analyses, but not in multivariate. E-cadherin, being responsible for epithelial cell junction, is rarely expressed in STS, except for synovial and epithelioid sarcomas, as well as mesothelioma, which naturally express both epithelial and mesenchymal markers. As could be expected, E-cadherin was in this study expressed aberrantly in a minority of STS and failed to demonstrate any association with survival.

NF-kB 1 (p50 and its precursor p105) is one of five members of the NF-kB family. These are transcription proteins responsible for control of inflammation, regulation of cell cycle and cell proliferation. NF-kB is constitutively activated in various tumor cells where it promotes cell proliferation, survival, metastasis, inflammation, invasion, and angiogenesis [39]. Its influence on tumorigenesis is rather controversial. Indeed, while the majority of the investigators confirm that this marker augments tumor invasiveness and metastasis resulting in shorter DSS, in a recent study by Al-Saad et al., NF-kB p 105 was reported to have a favourable impact on DSS in operable non-small cell lung carcinoma patients [40]. We found NF-kB p 105 expression in STS to indicate a poor prognosis.

Par-6 and PKC-ζ (one of four atypical PKCs) belong to the Par3/Par-6/aPKC polarity complex that governs diverse cell functions such as localization of embryonic determinants and establishment of tissue and organ during the embryonal period and regulation of cell polarity and the asymmetric division of cells in mature organisms [41]. Both Par-6 and PKC-ζ have been identified as EMT-associated biomarkers [42] and found to enhance proliferation, migration and invasiveness in cell cultures [43], [44]. We were unable to retrieve studies on Par-6 expression in human tumor tissue through PubMed searches. Cornford et al. reported that PKC-ζ expression was significantly higher in prostatic carcinomas than in non-neoplastic prostate tissue [45]. In STS, we observed that PKC-ζ expression was a significant indicator of shorter DSS.

Vimentin is an acknowledged marker of higher aggressivity in epithelial tumors. Its negative influence on patient survival has been demonstrated in several human cancers including breast [46], gastric [47] and oral squamous cell carcinoma [48]. The STSs which by definition express vimentin are not generally investigated for the prognostic importance of its grade of expression. In our material, all tumor cells were positive for vimentin, but at varying degrees. All STSs were dichotomized as strongly positive tumors or not, but there was no difference in survival between these two groups of patients.

In conclusion, we have characterized the STS phenotype with respect to tumor aggressiveness and DSS. We found also that all the tumors included in the non-GIST STS group shared this phenotype at different degrees. Moreover, our findings are in agreement with results of a number of studies that have investigated the roles of these markers in other, especially epithelial, tumors. This makes us to believe that the processes we have explored in the study are universal and are not a feature of one or several distinct entities.

Although the precise molecular interactions resulting in STS tumor cell dedifferentiation are still unclear, our findings may help to identify a subgroup of patients with aggressive tumors which require adjuvant therapy. Moreover, the biomarkers indicating such aggressiveness can represent molecular targets with the future development of small-molecule targeted therapy.
